# Oleate of Manganese

**Published:** 1885-09

**Authors:** Franklin H. Martin

**Affiliations:** Fellow of the Chicago Gynecological Society; 2139 Wabash Ave.


### OLEATE OF MANGANESE. *By* FRANKLIN H. MARTIN, M. D.

*Fellow of the Chicago Gynecological Society.*

(Read before the Chicago Medical Society, August 3rd, 1885.)

There is little doubt left in the minds of therapeutists in regard to the value of manganese as a remedy in certain forms of menstrual trouble. The remedy, in the form of the permanganate of potash, was first brought to the attention of the profession by Ringer and Murrell of London in the spring of 1883. They recommended the drug in functional amenorrhœa. Soon after their article appeared iu the London *Lancet,* I commenced experimenting with the same preparation, and published the result of my investigation in the *New York Medical Record* for September 29th, 1883. That was the first, to my knowledge, that anything on the subject was published in this country.

In the course of my experiments, acting upon the theory that the drug produced menstruation by stimulating the menstrual organs, I was induced to give the remedy in menorrhagia, and metrorrhagia dependent upon an *atonic* condition of these organs. I found to my gratification that it acted equally as well in these conditions as in the opposite. In my published reports I have always been careful to emphasize this point, because it is a demonstration of the manner of action of the drug, *i. e.* by *stimulating* the menstrual organs. I have had unmistakable evidence of its action as a menstrual stimulant, in amenorrhœa, menorrhagia and metrorrhagia. I have obtained very gratifying results from its administration in the irregularities incident to approaching menopause. I have also received very gratifying letters from a large number of physicians in this country, containing confirmatory proof of its efficacy in one or more of the several conditions mentioned above. The remedy has been favorably commented upon by THOMAS of New York, who said: "I think it is the best emmenagogue which has yet been discovered." Dr. Roberts Bartholow not only recognizes its applicability in amenorrhœa, but also its power as a general menstrual stimulant, making it equally efficacious in other forms of menstrual difficulties, dependent upon an *atonic* condition. He said: "The same power which can so stimulate the sexual functions must, when exerted in other directions, prove equally effective."

After publishing my second report on this subject, I received a letter from Sydney Ringer, of London, in which he expresses his gratification at the result of my experiments, and in which he says: "Like you, I have found the permanganate most useful in *atonic* conditions." And further, he says: "I was quite prepared to learn that the permanganate is useful in menorrhagia.

Since there is no longer any doubt about the great value of manganese in these distressing menstrual difficulties, the next formidable problem for the therapeutists to solve, is—how shall it be administered? The permanganate of potash, the original preparation used for experiments, and administration, in any form, is liable to act as an irritant to the stomach, and is very objectionable to many on that account. It has in many ingenious ways been made into pills, but as these pills must of necessity have for their basis kaolin, or some other inorganic substance, they are not satisfactorily dissolved and absorbed. The compressed tablets of Wyeth & Brother, and others, are objectionable in many cases of irritable stomach, because of the irritating undiluted drug coming in direct contact with its mucous membrane. The binoxide of manganese has, on account of its insolubility, proved to be inert. Therefore because of the many difficulties of administration this valuable remedy has not met with the reception by the profession that is its due.

While having this fact under consideration, it was suggested to me by Dr. Lewis L. McArthur, of this city, that an oleate of manganese might be prepared. Inasmuch as the method of prescribing other popular drugs in the form of oleate is becoming quite universal, the scheme appeared feasible. An oleate of manganese, was prepared for me by Mr. Edward Kreyssler, a very competent chemist with the firm of Forysth & Schmid, of this city. I am indebted to Mr. Kreyssler for the following account of the *method of preparation, and of its physical and chemical properties.* The oleate of manganese was prepared as follows: A solution of sulphate of manganese was made in distilled water, and to it a solution of sodium oleate was added. On mixing these two solutions gradually, and with constant stirring, a precipitate of oleate of manganese resulted. This precipitate, upon heating, changed to a putty-like mass. This was washed several times with warm distilled water, to remove the sodium sulphate, and the resulting putty-like mass was the pure oleate of manganese.

It is of a light gray color, having a pinkish hue, of a sweet musty taste, and peculiar clay-like odor. It is sparingly soluble in alcohol, and soluble in ether, chloroform, olive oil, and oleic acid.

THE METHOD OF APPLICATION.—Of a twenty per centum solution of the oleate in oleic acid, one half of a drachm to a drachm is applied to the abdomen of the patient, and its absorption promoted by friction, produced by vigorous rubbing of the surface with the palm of the hand or with the fingers. The rubbing should be continued until the oil has entirely disappeared by absorption.

In cases where it is found impracticable to apply it to the abdomen on account of tenderness, it may be applied to the back or the inner surfaces of the thighs.

In *amenorrhœa* it should be applied, if possible, every night for a week preceding the expected menstrual period, or at the time the menstruation is due, and until it makes its appearance. In cases of *menorrhagia* or *metrorrhagia* it can be applied in smaller quantities, every night, until the desired effect is produced.

REPORT OF CASES.—At the present writing, I have but four cases to report. I have prescribed the preparation in fifteen (15) cases, all but four (4) have as yet failed to report to me.

In the four that I have been able to keep under observation, the result has been very gratifying. Two of the cases were amenorrhœa in young women, one irregular menstruation in early married life, one irregularity incident to approaching menopause.

CASE 1. Had passed two menstrual periods and the remedy was prescribed about one week after the last menstruation was due. On account of abdominal tenderness the oil was applied to the back. One drachm of a twenty per centum solution was applied for three consecutive nights, at the end of which time menstruation appeared and lasted four days.

CASE 2. Was that of a young girl 19 years of age, whose menstrual flow had always been irregular. The cause I attributed to an over-worked nervous system, the young lady having just passed a long anxious siege of college examinations. She had passed two or three menstrual periods without a show, and had lost account of when the next period was due. The oleate was prescribed. It was applied four nights to back and abdomen, by a competent nurse, when the most natural menstruation the young lady had ever experienced commenced.

CASE 3. A married woman, age 42, who had always been healthy until six months ago. Since that time she has been suffering with irregular menstruation. At one time it did not appear for two months, the suffering from "pressure" in the meantime being very severe. On the other hand, at times the flow had been excessive, and the result was almost complete exhaustion. At the time the oleate was prescribed, the patient was suffering from distressing backache, headache and pelvic pressure, from suppression. At this time her menstruation, was two weeks over due. A drachm of the twenty per centum solution of the oleate was used two nights, thoroughly applied by a nurse, when the flow appeared suddenly, and all the distressing symptoms of former menstruations were absent.

CASE 4. A dispensary patient, married, age 25 years, had had no children. She had not menstruated for two and one-half months. No signs of pregnancy further than the suppression of the menses. The patient was poorly nourished. She had "caught cold." By physical examination nothing was found to account for this condition of affairs, except a small, and poorly developed uterus. The patient was ordered to use the oleate, and directed how to use it. She returned in one week menstruating. The flow had appeared three days after using the medicine.

2139 Wabash Ave.

